# A common denominator: PTSD, rapid eye movements, and fear extinction

**DOI:** 10.1073/pnas.2511191122

**Published:** 2025-08-04

**Authors:** Chika Edward Uzoigwe

**Affiliations:** ^a^Department of Medicine and Surgery, Harcourt House, Sheffield S10 1DG, United Kingdom

The work of Zhang et al. is at the fulcrum of a number of lines of research (1). There is a missing piece in the physiological puzzle the authors must report. The authors observe that dopaminergic neuronal stimuli from the ventral tegmental area (VTA) to the basolateral amygdala (BLA) are germane to fear extinction and thus potentially its impediment in conditions such as posttraumatic stress disorder (PTSD). However, the VTA to BLA neuronal route is the exact same pathway that elicits rapid eye movements in rapid eye movement (REM) sleep (2). This is critically important. In the treatment of PTSD, a current therapy known as eye movement desensitization and reprocessing therapy involves encouraging the participant to effect bilateral eye movements, akin to those during REM sleep while focusing on the distressing experience (3). It has been shown to be effective and is recommended by World Health Organisation and United Kingdom National Institute for Health and Care Excellence; however, the exact mechanism remains unclear (4). REM sleep is critical to processing traumatic events (5). Zhang et al. show a potential neuronal basis for this. It is important to determine from the study if the neuronal stimulation resulted in any increase or change in eye movements in the murine subjects. This information would be instrumental in the further elucidation of the mechanisms that belie fear extinction and pathologies and therapeutic interventions thereof.
